# Bridging the Gap: Connecting the Mechanisms of Immune-Related Adverse Events and Autoimmunity Through PD-1

**DOI:** 10.3389/fcell.2021.790386

**Published:** 2022-01-03

**Authors:** Adam Mor, Marianne Strazza

**Affiliations:** ^1^ Columbia Center for Translational Immunology, Columbia University Medical Center, New York, NY, United States; ^2^ Division of Rheumatology, Department of Medicine, Columbia University Medical Center, New York, NY, United States

**Keywords:** immunotherapy, PD-1, immune checkpoint inhibitors, T cells, immune-related adverse events, autoimmunity

## Abstract

The emergence of anti–cytotoxic T-lymphocyte antigen 4 (anti-CTLA-4), anti–programmed cell death 1 ligand (anti–PD-1), and anti–PD-L1 antibodies as immune checkpoint inhibitors (ICIs) revolutionized the treatment of numerous types of tumors. These antibodies, both alone and in combination, provide great clinical efficacy as evidenced by tumor regression and increased overall patients’ survival. However, with this success comes multiple challenges. First, while patients who respond to ICIs have outstanding outcomes, there remains a large proportion of patients who do not respond at all. This all-or-none response has led to looking downstream of programmed cell death 1 (PD-1) for additional therapeutic targets and for new combination therapies. Second, a majority of patients who receive ICIs go on to develop immune-related adverse events (irAEs) characterized by end-organ inflammation with T-cell infiltrates. The hallmarks of these clinically observed irAEs share many similarities with primary autoimmune diseases. The contribution of PD-1 to peripheral tolerance is a major mechanism for protection against expansion of self-reactive T-cell clones and autoimmune disease. In this review, we aim to bridge the gaps between our cellular and molecular knowledge of PD-1 signaling in T cells, ICI-induced irAEs, and autoimmune diseases. We will highlight shared mechanisms and the potential for new therapeutic strategies.

## Introduction

Day to day, T cells encounter and attack cancer cells as foreign, effectively preventing tumor growth. When cancer cells evolve under this selective pressure and develop mechanisms to escape T-cell immunity, tumors are able to grow. This spawned the idea that T-cells can be the target of therapeutics to enhance their ability to target cancer cells. In this way, external intervention can tip the scale back in favor of the immune system, thereby limiting tumor growth and even causing tumor regression.

Historically, promoting T-cell activation and recognition of tumors was a sought-after mechanism for tumor intervention and drug design (Rosenberg, 2014). When inhibitory immune checkpoints were discovered, starting with cytotoxic T-lymphocyte antigen 4 (CTLA-4), the strategy for these interventions shifted to focus on blocking the function of these coinhibitors (Allison et al., 1995; Boussiotis, 2016). PD-1 is one such coinhibitor expressed on T-cells and exploited by tumor cells as a means of evading immune detection. By overexpressing programmed cell death 1 ligand (PD-L1), a PD-1 ligand, tumor cells engage PD-1 on T-cells, blocking activation and function. This pathway has been effectively targeted by monoclonal antibodies (Abs) targeting PD-1 itself or PD-L1 with great success in many patients (Pedoeem et al., 2014).

Despite the success, however, there remain numerous challenges to immune checkpoint inhibitors (ICIs) use that must be met head on in order to best advance into the next generation of therapeutic strategy. These challenges include increasing responsiveness to PD-1 and CTLA-4 blockade, uncovering new targets to optimize pathway blockade, and predicting and effectively managing adverse events. ICIs are associated with the development of immune-related adverse events (irAEs) that affect various tissues and organ systems throughout the body, although usually not more than one in each patient (Sandigursky and Mor, 2018). These acute or chronic inflammatory responses are thought to emerge as a result of unchecked T-cell activation and loss of tolerance. irAEs share many hallmarks of organ-specific autoimmunity, including the expansion of self-antigen recognizing T-cell receptor (TCR) clones. These observations led to the study of PD-1 agonists in the treatment of autoimmunity.

In this review, we will delve into the PD-1 signaling pathway in T-cells to highlight the mechanism of action of ICIs, the cellular functions altered by the PD-1 cascade, and the emerging understanding of irAEs resulting from the use of checkpoint inhibitors and aim to bridge the gap between the use of ICIs, irAEs, and primary autoimmunity. Through understanding the PD-1 signaling pathway, we as a field can more effectively modulate T-cell function to improve clinical outcomes.

### PD-1 Biology

PD-1 is expressed by T-cells, among other hematopoietic cells, whereas its ligands PD-L1 and PD-L2 are more differentially expressed (Pauken et al., 2021). PD-L2 is generally expressed by professional antigen-presenting cells including macrophages and dendritic cells (DCs). PD-L1 expression can be found more broadly throughout the body. PD-L1 is constitutively expressed on T-cells, B-cells, DCs, and other myeloid-derived cells, as well as on cells that are nonhematopoietic in origin. Often, PD-L1 expression is upregulated in tumors of varying origins.

Under homeostatic conditions, PD-1 is essential for the maintenance of peripheral tolerance. It functions in this capacity along with CTLA-4 through upregulation of expression at the cell surface following antigen stimulation of the TCR. Tyrosine phosphorylation is a key component of PD-1 signaling, due in part to the immunoreceptor tyrosine-based inhibition motif and immunoreceptor tyrosine-based switch motif within the intracellular tail, which become phosphorylated following ligand binding and recruit the phosphatases Src homology 2 domain-containing protein tyrosine phosphatase 1 and 2 (SHP1 and SHP2) to dephosphorylate TCR signaling mediators. In general, this leads to inhibition of numerous T-cell functions including adhesion, proliferation, and cytokine secretion. There is mounting evidence that this is an incomplete picture of what occurs downstream of PD-1 ligation, perhaps shedding light on the incomplete and unexplained responsiveness to PD-1 blockade in the clinic (Riley, 2009; Wang et al., 2021).

To start, the mechanism through which PD-1 ligation initiates SHP2 activation has not been fully elucidated. This phosphatase has been attributed to enzymatic function not only downstream of PD-1, but also downstream of the TCR itself, suggesting that the enzyme serves a role in both T-cell activation and inhibition (Chan et al., 2008). In addition, few substrates for SHP2 in T-cells have been identified (Pedoeem et al., 2014; Strazza et al., 2021a). In the capacity that SHP2 enhances T-cell activation, it has been shown to dephosphorylate inhibitory sites of positive regulators including AKT and ERK and activating sites of negative regulators including CSK, CRK, and PAG (Frearson and Alexander, 1998; Chemnitz et al., 2004; Bardhan et al., 2019; Celis-Gutierrez et al., 2019). Downstream of PD-1, the main evidence of a contribution of SHP2 function comes from the dephosphorylation of the coreceptor CD28 (Hui et al., 2017; Khan et al., 2021). Perhaps sublocalization and temporal segregation of signaling can best explain these dual, even opposing, roles for SHP2 (Valitutti et al., 2010). In the initial phase after TCR activation, SHP2 acts downstream of the antigen receptor to enhance signaling. Later, after PD-1 is upregulated at the cell surface and encounters its ligand, SHP2 is recruited to the intracellular tail of PD-1 where its substrates are mediators of T-cell inhibition. In our own recent study, we identified ITK tyrosine kinase as a substrate for SHP2 downstream of PD-1 ligation, but not the TCR (Strazza et al., 2021a). ITK is a member of the TEC family of kinases (Weeks et al., 2021) and is recruited to the vicinity of the TCR following antigen stimulation where it is phosphorylated at two tyrosines (Berg et al., 2005). Following PD-1 ligation, ITK is dephosphorylated by SHP2 (Strazza et al., 2021a). Although there are other SHP2 substrates, identified and unknown, downstream of PD-1 ligation ITK is one contributor to PD-1 inhibition of T-cell function (Strazza et al., 2021a).

PD-1 signaling goes beyond tyrosine phosphatase activation, and the field’s understanding of the phospho-landscape following PD-1 ligation has remained limited. To fill in some of these gaps and uncover new potential targets for intervention into this pathway, we combined isobaric labeling with phospho-peptide enrichment and mass spectrometry (Tocheva et al., 2020). In this study, we stimulated Jurkat T cells with anti-CD3 Abs, to stimulated the TCR pathway, in the presence or absence of recombinant PD-L2 for either 30 s or 5 min. We compared phospho-sites identified between anti-CD3 and anti-CD3 with PD-L2 for the 30 s and 5 min stimulations independently and found that PD-1 ligation increased serine and threonine phospho-sites by 53 phospho-sites at 30 s and by 105 sites after 5 min relative to anti-CD3 alone (Tocheva et al., 2020). We additionally observed increased tyrosine phosphorylation of four proteins when stimulation included PD-L2 (Tocheva et al., 2020). Of course, the results of this study also included a greater proportion of all three phosphorylation types at 30 s when PD-L2 was included in the stimulation. After 5 min of stimulation, the three phosphorylation types diverged into different trends, with the majority of captured serine and threonine sites following PD-1 ligation being up-phosphorylated and the majority of tyrosine sites with decreased phosphorylation (Tocheva et al., 2020). Through this study, we revealed that PD-1 signaling leads to both an increase and decrease in phosphorylation and, importantly, that these phospho-sites are not restricted to tyrosine residues. Based on functional enrichment analysis, we were able to conclude that the increased phosphorylation of proteins following 5 min of stimulation including PD-L2 was involved in the negative regulation of gene expression and protein translation (Tocheva et al., 2020).

With regard to T-cell function, we and others have shown that engaging PD-1 in combination with TCR activation leads to inhibition of cytokine secretion, cell adhesion, cytotoxicity, and proliferation at the whole population level (Pedoeem et al., 2014; Azoulay-Alfaguter et al., 2015). Single-cell analysis has provided evidence of differential signaling and function across different effector maturation states or T-cell subsets, to the extent that there is evidence that PD-1 may activate individual T-cell functions in certain contexts (Riley, 2009; Strazza et al., 2021b). For example, both proliferating and nonproliferating cytotoxic CD4 T-cells isolated from tumors have been shown to have a gene signature associated with response to anti–PD-1 (Oh et al., 2020). We have also shown that in response to TCR stimulation in combination with PD-L1 and PD-L2, a population of CD4 and CD8 T-cells becomes hyperproliferative, undergoing an additional population doubling compared with TCR stimulation alone (Strazza et al., 2021b; Lerrer et al., 2021). Isolating this hyperproliferative population and comparing the transcriptional signature to the proliferation inhibited population of cells uncovered extensive differences, including the enrichment of genes associated with T-cell activation and cellular adhesion in the hyperproliferating population (Strazza et al., 2021b). In addition, genes associated with the negative regulation of T-cell activation and the cell cycle were enriched in the nonproliferating population (Strazza et al., 2021b). These gene set differences suggest that despite activation of the PD-1 signaling pathway, the observed increased proliferation is an active process mediated by positive signaling events. This is additionally evidenced by the prevalence of central memory (T_CM_) and effector memory (T_EM_) in hyperproliferating CD8 T-cells (Lerrer et al., 2021). The stimulation of naive T cells through the TCR results in cell maturation toward a T_CM_ phenotype, and as expected, proliferating T-cells stimulated through the TCR alone are predominantly T_CM_. Strikingly, the addition of PD-1 caused further maturation with a prevalence of T_EM_ cells within the hyperproliferating population (Lerrer et al., 2021). RNA sequencing (RNAseq) analysis of naive, T_CM_, and T_EM_ cell types individually stimulated through the TCR in the presence or absence of PD-1 ligation reveals that more genes were differentially regulated among the cell types (Lerrer et al., 2021).

In combination with TCR activation, PD-1 ligation has also been shown to have differential effects on cytokine secretion (Rahimi Kalateh Shah Mohammad et al., 2020; Adorisio et al., 2021; Strazza et al., 2021b), with interleukin 2 (IL-2) being inhibited by PD-1 ligation, whereas IL-15, a cytokine that is structurally and functionally very similar to IL-2, is unaffected. By looking again at the isolated hyperproliferative T-cell population, IL-15 was enriched in the nonproliferating population (Strazza et al., 2021b), perhaps explaining why the net effect on a heterogeneous T-cell population would be observed as no difference. These observations and conclusions deviate from the expectation that PD-1 signaling will lead to the inhibition of the production of all cytokines that enhance immune cell activation.

T follicular helper (T_FH_) cell-associated genes and proteins were enriched within the hyperproliferating population of cells stimulated through the TCR and PD-1 (Shi et al., 2018; Strazza et al., 2021b). In addition, naive cells that were stimulated through the TCR and PD-1 expressed higher levels of T_FH_-associated genes relative to unstimulated and TCR stimulation (Lerrer et al., 2021). Single-cell RNAseq (scRNAseq) of melanoma samples before and after pembrolizumab administration, an anti–PD-1 Abs, revealed that the majority of T_FH_ cells were isolated from patients who had a positive response to pembrolizumab posttreatment and that these cells were present in the same patients pretreatment (Sade-Feldman et al., 2018; Strazza et al., 2021b). This is particularly interesting in that a portion of patients who receive ICIs are observed to have accelerated tumor growth posttreatment (Borcoman et al., 2019), referred to as hyperprogressive disease, through a largely unknown mechanism (Shen et al., 2021). Through scRNAseq of tumors from patients with hyperprogressive disease pretreatment and posttreatment, it was found that the tumors became less immunogenic through treatment, and an enrichment of ILC3 cells, a subset of innate lymphoid cells, was observed (Xiong et al., 2018). Overall, signaling downstream of PD-1 ligation in T-cells is far more complex than was initially appreciated, and this may provide a mechanism for the observed activation of some cell types or functions.

### Targeting PD-1 to Treat Cancer

#### Anti–PD-1 Abs

The 2018 Nobel Prize in Physiology and Medicine was awarded to Tasuku Honjo and James Allison for their discoveries of PD-1 and CTLA-4, respectively, and the impact these discoveries made on clinical cancer care (Smyth and Teng, 2018). Suppression of T-cell activation by PD-1 and CTLA-4 is considered a major escape mechanism of cancer cells, and inhibition of these proteins by ICIs successfully activates the immune system to identify and eliminate cancer cells. ICIs have brought a breakthrough in cancer immunotherapy, reviving the hope of curing patients with end-stage cancer, including a wide variety of cancer types.

The first ICIs developed to target PD-1 were neutralizing monoclonal Abs. Anti–PD-1 and anti–PD-L1 Abs are highly effective with prolonged overall survival, and additional Abs are in development and showing great promise in clinical trials. The anti–PD-1 Ab, pembrolizumab, was developed by Merck and approved by the Food and Drug Administration (FDA) in 2014 to treat melanoma (Koulouris and Mountzios, 2021). Shortly after, it became the first immunotherapy drug approved for use based on the genetic mutations rather than anatomical site of the tumor. It was demonstrated that patients with higher mutation burden respond better to this intervention. Both objective responses and progression-free survival were shown to be higher than in patients with low mutation burden. The second anti–PD-1 Ab, nivolumab, was developed by Bristol Myers Squibb and approved by the FDA in 2014 to treat advanced melanoma, squamous cell lung cancer, renal cell carcinoma (RCC), and Hodgkin lymphoma (Sanaei et al., 2021). Next, cemiplimab was developed by Regeneron and was the third anti–PD-1 Ab to gain approval from the FDA in 2018 for the treatment of cutaneous squamous cell carcinoma (Ahmed et al., 2019). Spartalizumab is a PD-1 inhibitor Ab developed by Novartis to treat both solid and hematopoietic malignances, which as of 2019 has entered phase 3 clinical trial (Even et al., 2021). Sintilimab is a human anti–PD-1 Ab that has been developed by Eli Lilly for patients with non–small cell lung carcinoma (NSCLC) (Zhou et al., 2021). Dostarlimab is a humanized Ab against PD-1 under investigation by GlaxoSmithKline. Tislelizumab and toripalimab are humanized immunoglobulin G4 (IgG4) anti–PD-1 Abs in advance stages of clinical trials. Neutralizing Abs targeting PD-L1 (atezolizumab, avelumab, and durvalumab) have been approved by the FDA to treat multiple types of cancers (Sanaei et al., 2021).

#### Combination Therapy

Similar to other biologics, combinations of ICIs have been evaluated for multiple clinical indications and have improved clinical efficacy over monotherapy strategies. Simultaneous PD-1 and CTLA-4 blockade (nivolumab and ipilimumab) is farthest along in standard clinical use (Jiang et al., 2021; Sanaei et al., 2021). In patients with metastatic melanoma and progressive NSCLC, this combination improved survival compared with ipilimumab monotherapy or chemotherapy, respectively. In patients with RCC, the combination improved survival compared with sunitinib, small-molecule multitargeted receptor tyrosine kinase inhibitor (Amin et al., 2018; Ikeda et al., 2021). This combined therapeutic approach also has clinical efficacy in hepatocellular carcinoma and microsatellite instability-high and mismatch repair–deficient colorectal cancer (Finn et al., 2020; Lichtenstern et al., 2020).

The success of combined treatment with anti–PD-1 and anti-CTLA4 does come with challenges. As discussed below, the rate of irAEs was higher with combined nivolumab plus ipilimumab versus nivolumab or ipilimumab alone. The combination of anti–PD-1 Abs with agents targeting epidermal growth factor receptor and anaplastic lymphoma kinase has been associated with life-threatening irAEs in patients with advanced NSCLC (Lindeman et al., 2013; Spigel et al., 2018; Cheng et al., 2019; Xing et al., 2019). Notably, in patients with metastatic melanoma, PD-1 blockade and BRAF plus MEK inhibitors have been safely combined and have regulatory approval in this setting (Zaremba et al., 2021).

### Immune-Related Adverse Events

**FIGURE 1:**
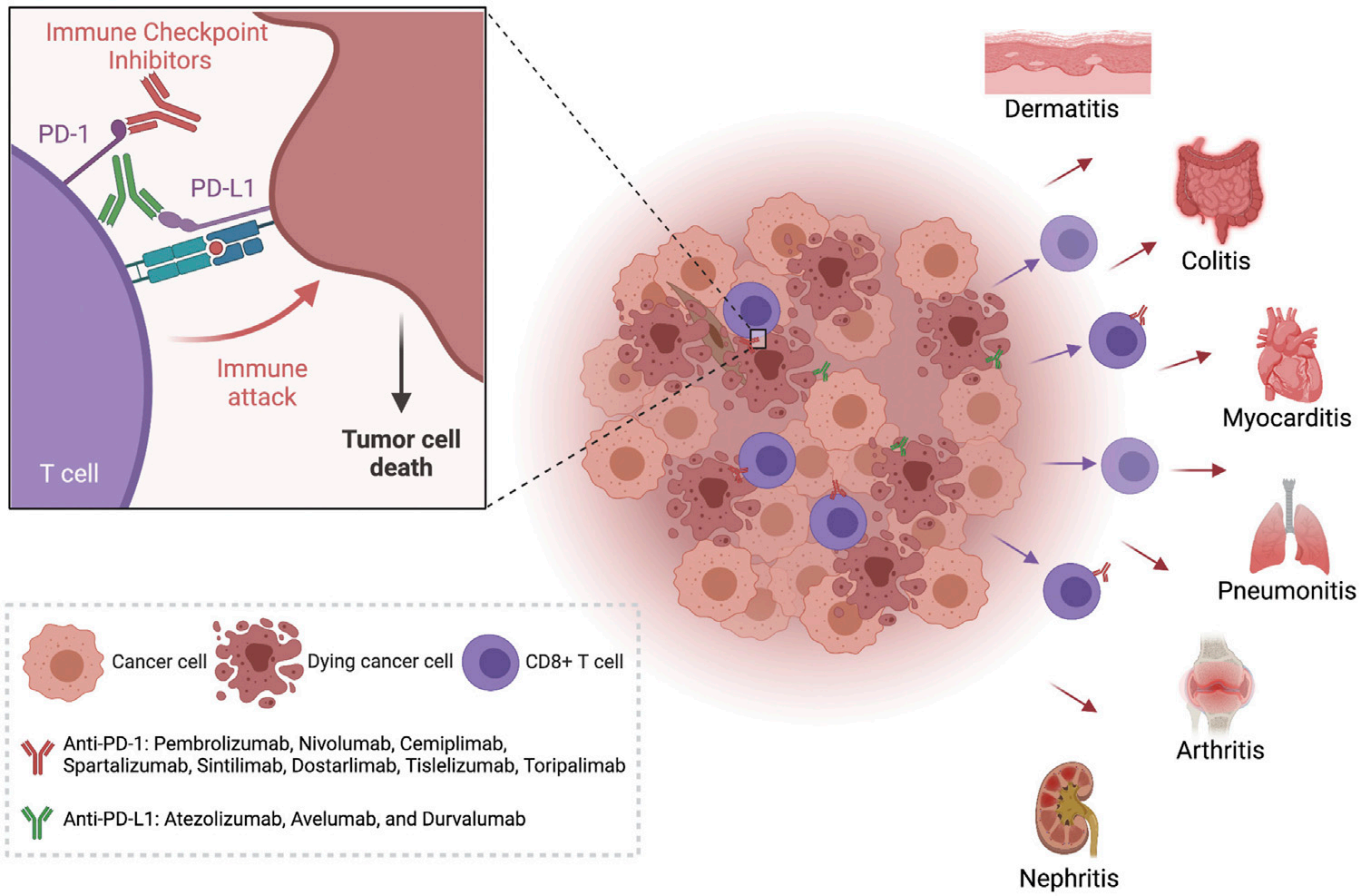
Immune Checkpoint Inhibitors can lead to the development of inflammation in many organs. The treatment of tumors with anti-PD-1 or anti-PD-L 1 antibodies increases the activation of tumor infiltrating cytotoxic T cells and leads to tumor cell death. These activated T cells with decreased function of the PD-1 checkpoint pathway have also been observed in peripheral circulation and have been isolated from inflamed organs in patients with immune related adverse events (irAEs). Created with Biorender.com.

irAEs that develop from ICIs are a broad array of side effects affecting different organ systems, including dermatologic, gastrointestinal, hepatic, endocrine, pulmonary, musculoskeletal, and other less common inflammatory events (Figure 1). Given that ICIs block inhibitory signaling pathways in T-cells, the nonspecific enhancement of immune response induced by these mediators can also directly attack normal tissues, thereby facilitating autoimmune and autoinflammatory responses against many organs (Sandigursky and Mor, 2018). Compared with toxicities resulting from chemotherapy, irAEs develop at any time during the treatment, even months after discontinuing the treatment with the checkpoint blockade (Choi and Lee, 2020). In addition, as the onset of irAEs is often sudden, and even fatal toxicities may occur, it is essential that clinicians recognize and manage the events early (Alrabadi et al., 2021).

Although irAEs differ according to the type of the Abs used, there are common clinical features (Sosa et al., 2018). First, irAEs tend to be organ-specific rather than involving patients’ multiple organs at the same time. Second, toxicity onset could be delayed, in some cases, months into the ICIs treatment. Third, toxicity following ICIs is not apparently associated with the dose, and therefore, dose reductions will not prevent them from happening. Because of this, irAEs frequently require permanent treatment discontinuation. These events are particularly challenging in the adjuvant setting, as late-onset toxicities may induce permanent damage in patients who might had been otherwise already cured of their cancer.

Within the literature, there are multiple ways to categorize irAEs (Sosa et al., 2018; Chhabra and Kennedy, 2021). Acute irAEs are observed during treatment, delayed irAEs emerge after the completion of treatment, and chronic irAEs are defined as lasting more than 12 weeks beyond termination of treatment (Patrinely et al., 2021). Organ involvement usually correlates differentially with time of onset, with dermatologic, gastroenterological, hepatic, and respiratory observed to be more acute and neurologic, endocrine, renal, and musculoskeletal more often chronic (Troxell et al., 2016; Zhong et al., 2021a). Overall, chronic irAEs are more difficult to treat and will require long-lasting immunosuppression. The frequency of occurrence of irAEs has also been used to establish categories, those more frequently reported (dermatologic, gastrointestinal, endocrine, respiratory, and rheumatologic/musculoskeletal) and uncommon (cardiovascular, hematologic, renal, neurologic, and ophthalmologic) (Puzanov et al., 2017). As a field, we are beginning to understand that involved organs differ by the underlying mechanism mediating the effect.

Gastrointestinal irAEs include mucositis, aphthous ulcers, gastritis, abdominal pain, and colitis. ICI-induced colitis occurs most frequently following combined inhibition of PD-1 and CTLA-4, with some reporting incidence of up to 32% (Zhang et al., 2018; Najjar et al., 2020; Portenkirchner et al., 2021). Interestingly, the rate of incidence may be influenced by the type of cancer being treated (Wang et al., 2017a; Portenkirchner et al., 2021). On histological examination, the features of ICI-induced colitis are similar to acute colitis with cryptitis, intraepithelial neutrophilic lymphocytes, mucosal ulcerations, crypt abscesses, and apoptosis (Berman et al., 2010; Verschuren et al., 2016; Adler et al., 2018; Geukes Foppen et al., 2018; Bellaguarda and Hanauer, 2020; Chen et al., 2020; Hayashi et al., 2021; Portenkirchner et al., 2021). In patients with metastatic melanoma that were given ICI therapy that included anti–CTLA-4, an increased number if IL-17A–secreting CD4 T-cells (T_H_17) was observed in peripheral circulation of those who developed colitis (von Euw et al., 2009; Anderson et al., 2019). The management if ICI-induced colitis is primarily by steroids, although if the case is especially severe or life-threatening, then ICIs should be discontinued and other immune suppressive therapeutics should be administered (Stidham et al., 2014; Bergqvist et al., 2017; Abu-Sbeih et al., 2018; Wang et al., 2018; Mir et al., 2019). More research is necessary to fully understand the potential for targeting IL-17A due to the fact that the cytokine has been linked to both immunotherapeutic efficacy and tumorigenesis (Anderson et al., 2019).

Cardiovascular irAEs including myocarditis are rare, although the rate of incidence varies by report from 0.09% to 2.4%, depending on the cohort (Johnson et al., 2016; Sznol et al., 2017; Tajiri and Ieda, 2019). Strikingly, of those patients who experience myocarditis following combined PD-1 and CTLA-4 blockade, the mortality is greater than 60% (Johnson et al., 2016; Tajiri and Ieda, 2019). The treatment strategy for these patients remains quite loosely defined, with early steroid intervention used in most cases with the addition of other immunosuppressive agents, high-dose intravenous immunoglobulin therapy, plasmapheresis, and immunoadsorption therapy for nonresponding patients (Johnson et al., 2016; Tajiri and Ieda, 2019). As for clinical mechanism, we have learned from murine models that deficiency in CTLA-4 leads to the development of severe myocarditis with T-cell infiltration (Waterhouse et al., 1995; Tajiri and Ieda, 2019). Still, multiple mechanisms have been proposed, and further study is warranted (Johnson et al., 2016). The loss of PD-1 in mice is less straightforward, with genetic strain differences in cardiac involvement. MRL mice are an autoimmune prone strain of mice, and the loss of PD-1 on this background is associated with myocarditis with T-cell infiltration (Wang et al., 2010; Tajiri and Ieda, 2019).

Overall, the frequency of irAEs can be broken down by the type of ICIs received. A recent meta-analysis of the frequency of irAEs over multiple trials revealed that irAEs occurred in 74% of cancer patients treated with anti–PD-1 or PD-L1 Abs, 89% of patients treated with anti–CTLA-4, 90% of patients in the ICI combination group, and 89% of patients in the ICIs with chemotherapy group (Wang et al., 2017b). irAEs with grade 3 (severe) or grade 4 (life threatening) were reported in 14% of patients treated with PD-1 or PD-L1 inhibitors, 34% of patients treated with anti–CTLA-4 Abs, 55% of patients treated with ICI combinations, and 46% of patients treated with combinations of chemotherapy agents (Chhabra and Kennedy, 2021). The rates of irAEs leading to treatment withdrawal were 6% after using the PD-1 or PD-L1 inhibitors, 21% for anti–CTLA-4 Abs, 38% for ICI combinations, and 13% for combinations with chemotherapy (Sheng et al., 2021). These differential occurrences may hint at the underlying mechanisms and should be factored into the development of the next generations of ICIs.

#### Biomarkers of irAE Development

Biomarkers are being considered to predict the risk of developing irAEs and as an aid in the early identification of such complications (Zhong et al., 2021b). Examples include serum IL-17 (Tyan et al., 2021), eosinophilia (Baroz et al., 2021), and combined toxicity scores based on gene expression profiling of immunologically predictive cytokines. The optimal predictive biomarker remains to be defined (Martini et al., 2021).

Accordingly, to discover additional predicative biomarkers of irAEs, we recently undertook an scRNAseq analysis of circulating T-cells from cancer patients who develop irAEs following anti–PD-1 therapy. We aimed to uncover specific TCR sequences that predisposed the cancer patients to develop organ-specific toxicities and identify different populations of T-cells associated with diverse irAEs. We propose that quantification of these unique populations of cells could serve as a possible biomarker in patients before treatment is initiated to predict the risk of irAE development. Using the K-nearest-neighbor–based network graph drawing layout (KNetL) (Tang et al., 2021), our analysis suggests that low percentages of CD8 effector T-cells (T_EFF_) cells at baseline could predict irAEs arthritis, that more CD4 T_H_2 cells were associated with onset of pneumonitis, and that patients with thyroiditis had more CD4 T_H_17 cells before treatment with ICIs was initiated than those who did not develop this irAE (submitted manuscript).

#### irAE Treatment Strategies

The approach to manage irAEs is based on clinical experience, as no prospective trials have been conducted. The American Society of Clinical Oncology (Reynolds et al., 2021) and the Society for Immunotherapy of Cancer (Puzanov et al., 2017) organized multidisciplinary panels that reviewed the literature and proposed guidelines and organ-specific recommendations for the management of such toxicities (Puzanov et al., 2017; Brahmer et al., 2018). Treatment is based on the severity of the observed toxicity, within a defined 4-point grading: grade 1—mild, grade 2—moderate, grade 3—severe, and grade 4—life-threatening. First, all patients should be carefully monitored during treatment for initial evidence of grade 1 irAEs. If grade 2 irAEs are observed, the ICIs should be withheld and not be resumed until symptoms or toxicity is grade 1. If symptoms do not resolve within a week, the patient should be started on glucocorticoids. However, if the patient presents with grade 2 endocrinopathy, ICIs should be resumed only after hormone replacement is initiated. If a patient is experiencing grade 3 or grade 4 irAEs, ICI treatment should be immediately discontinued and high-dose steroids administered. In these cases, when symptoms lessen to grade 1, the glucocorticoids should be gradually tapered over a 1-month period. As others have also observed, it is our experience that symptoms will lessen within the first 3 days of glucocorticoid treatment if the patient will respond at all. When symptoms do not improve within these initial 3 days, glucocorticoid treatment can be discontinued, and anti–tumor necrosis factor α (TNF-α) can be administered (Dimitriou et al., 2021). As mentioned, when irAEs return to grade 1, ICIs can be resumed. A recent Canadian study focused on this population of patients undergoing ICI retreatment concluded that the same previously observed irAEs returned in 52% of patients (Hoa et al., 2021).

#### Preexisting Autoimmunity and Drug Rechallenge

A practical question is how to approach patients with preexisting autoimmune diseases prior to initiation of ICIs. Initial clinical trials excluded patients with preexisting autoimmune disease (Li et al., 2020), and for this reason, there are limited data available on responsiveness and development of irAEs in these patients (Weber et al., 2016; Reck et al., 2021). Most currently, available data do suggest that autoimmune disease does not greatly interfere with the safety of ICIs (Cappelli et al., 2017; Abdel-Wahab et al., 2018; Arbour et al., 2018; Schadendorf et al., 2019; Dolladille et al., 2020; Efuni et al., 2021; Reck et al., 2021), although a higher incidence of irAEs related to the initial autoimmune disease or worsening of the underlying autoimmune disease has been observed (Cappelli et al., 2017; Abdel-Wahab et al., 2018; Arbour et al., 2018; Dolladille et al., 2020; Li et al., 2020; Reck et al., 2021). Likewise, a higher risk for mild irAEs and discontinuation of ICIs have been observed in patients with underlying autoimmune diseases, whereas the risk and incidence of moderate to severe irAEs are not observed to be higher (Reck et al., 2021). No impact on ICIs efficacy and tumor clearance has been reported in patients on glucocorticoids or other immunosuppressive therapeutics, and objective response rates and overall survival are reported to be similar in patients with and without autoimmune diseases (Reck et al., 2021), although additional studies of efficiency of ICIs efficacy in this population are warranted (Bhatlapenumarthi et al., 2021).

#### Mechanism Underlying the Development of irAEs

The majority of the patients who receive treatment with ICIs develop irAEs, which often can be considered autoimmunity (Khan and Gerber, 2020). As such, understanding the mechanisms that control the breakdown of immune tolerance remains a critical goal. These mechanisms lead to excessive end-organ inflammation, and knowledge of their function is crucial not just for the identification of the predictive biomarkers for inflammatory toxicities, but also for the development of new generation of safer ICIs. The fact that there are different frequencies of irAEs across multiple ICIs suggests that there may be multiple cellular and molecular mechanisms of action.

CTLA-4 controls immunologic responses at early stages of T-cell activation, whereas PD-1 acts at later stages, limiting T-cell activity in the peripheral tissues. Therefore, anti–CTLA-4 Abs take effect by enhancing T-cell priming, whereas PD-1 blockade is thought to act by reinvigorating preexistingCD8 T-cell responses (Lee et al., 2015a). These differences can partly explain the increased frequency and severity of irAEs associated with anti–CTLA-4 Abs compared with anti–PD-1 Abs.

It is established that negative selection of T-cells does not eliminate all self-reactive cells and that these cells can be detected in the circulation. Peripheral regulatory T-cells (T_REG_) and surface expression of immune checkpoints prevent activation and expansion of these remaining self-reactive T-cells. As expected, this control is lost in cancer patients treated with anti–PD-1 Abs. A recent report of cancer patients who developed type 1 diabetes following anti–PD-1 Ab treatment confirmed the presence of islet-specific CD4 and CD8 T-cells in the peripheral blood of these patients (Mourad et al., 2021). Mechanistically, PD-1 signaling contributes to the cell-intrinsic inhibition of proliferation and pancreas infiltration of islet-reactive T-cells (Mourad et al., 2021). With regard to T_REG_ in this pathogenesis, it is yet to be fully understood if inhibition of the PD-1 pathway in T_REG_ leads to escape and activation of autoreactive islet-specific T-cells or if PD-1 blockade is more directly impacting the autoreactive cell population (Mourad et al., 2021). Similarly, increased infiltration of CD8 T-cells and reduced T_REG_ were reported following anti–PD-1 therapy in patients developing inflammatory myocarditis (Grabie et al., 2019). A role for PD-1 in protecting against T-cell-mediated myocarditis has been established through genetic models with PD-1 depletion in CD8 T-cells (Tarrio et al., 2012).

Additional evidence in support of the role of autoreactive T-cells in the pathogenesis of irAEs is the fact that early increased diversity of T-cell repertoire after ICI therapy correlated with the development of irAEs (Nakamura, 2019). In four cases of pneumonitis after treatment with ICIs, the T-cell repertoire in the inflamed lung lesions and tumors overlapped significantly, suggesting that cross-reactive T-cells against the tumors and a related antigen in normal tissue might be involved in irAEs pathogenesis (Zhang et al., 2021a). Perhaps surprisingly, these shared TCR sequences between tumors and inflammatory sites of irAEs are not restricted to one tissue. NSCLC patients who received anti–PD-1 Abs and developed skin irAEs were found to have shared TCR sequences in tissue samples isolated from both the primary tumor and the inflamed skin (Berner et al., 2019). Furthermore, these TCR sequences recognized tumor antigens that were shared with the inflamed skin (Berner et al., 2019). The presence of shared neoantigens arising across tumors also provides an explanation for shared irAEs across different cancers (Ward et al., 2016). Collectively, these studies point to the fact that T-cell clones responding to self-antigen are a major driving mechanism underlying the development of irAEs (Lee et al., 2021).

Though it must also be noted, that B cells and Abs also likely contribute to irAEs onset and progression (Willsmore et al., 2020; Lee et al., 2021). Cells of the innate immune system may be involved in the pathogenesis of irAEs as well, as evidenced through the use of whole-blood gene-expression profiling in melanoma patients treated with ICIs, to note that the neutrophil markers CD177 and CEACAM1 were associated with gastrointestinal irAEs (Pavan et al., 2019). Apart from immune cells, cytokines are also important regulators of host immune activity. Lim et al., analyzed the expressions of 65 cytokines in longitudinal plasma samples collected prior to and during ICI treatment of melanoma patients. The study concludes that eleven cytokines were significantly upregulated in patients who experienced severe irAEs (Lim et al., 2019). Furthermore, the investigators combined these cytokines into a toxicity score that was able to effectively predict the occurrence of irAEs. Notably, serum levels of IL-6, IL-17, and sCD163 are significantly associated with irAEs in cancer patients treated with ICIs (Lim et al., 2019).

Altogether, the potential mechanisms underlying the development of irAEs are still poorly elucidated and may be related to a combination of genetic predisposition, environmental triggers, and preexisting inflammation. The mediators of irAE pathogenesis seem to be equally diverse, with contributions from adaptive and innate immune cells, as well as circulating cytokines. Whether irAEs are representative of *de novo* events or indicative of underlying immune-mediated diseases is also unclear. Earlier studies suggest that irAEs correlate with improved response rates and long-term survival, whereas more recent studies failed to demonstrate such association (Hussaini et al., 2021; Zhao et al., 2021). Further studies are required to confirm whether experiencing an irAEs is predictive of anticancer treatment outcomes.

### PD-1 and Autoimmunity

As this review has already been discussed, the immune checkpoints, including PD-1, function to maintain peripheral tolerance, preventing self-antigen–driven inflammatory responses and autoimmunity. Any genetic mutations in PD-1, PD-L1, or PD-L2 that alter expression or binding have been associated with clinical autoimmunity (Tocheva and Mor, 2017). In animal models of PD-1 deletion, the exact phenotype varies, depending on the strain background, although all instances of PD-1 loss of function are associated with autoimmune development. While PD-1 knockout BALB/C mice develop autoimmune cardiomyopathy, PD-1 knockout C57BL/6 mice develop late-onset lupus-like disease (Paluch et al., 2018).

The observations from these animal models are recapitulated in a human population with regulatory polymorphisms in the *PDCD1* gene, which codes for PD-1, which has increased incidence of systemic lupus erythematosus (SLE) (Prokunina et al., 2002), atopy, and rheumatoid arthritis (RA) (James et al., 2005; Lee et al., 2015b) and progression in multiple sclerosis (MS) (Kroner et al., 2005). Sera samples from patients with RA were found to have elevated levels of PD-L1 Abs, and these levels correlated with disease severity (Dong et al., 2003). In addition, the success of interferon β (IFN-β) in the treatment of MS has been attributed to the upregulation of PD-L1 by myeloid cells (Schreiner et al., 2004). Collectively, this evidence lends support to the notion that engaging inhibitory checkpoints, such as PD-1, by ligands or agonists is a promising therapeutic strategy in the treatment of autoimmune diseases.

#### Rheumatoid Arthritis

Although the autoimmune disease RA is a chronic inflammation centered around the joints and synovium, it is truly a systemic disorder. At the primary site, the joint, the chronic inflammation leads to destruction of the cartilage and bone. Throughout the body, numerous organ systems are impacted, including the cardiovascular, pulmonary, and digestive systems. As mentioned, interference with the PD-1 pathway can accelerate disease progression, and for this reason, it is considered to be a protective pathway in this disease.

Murine models of RA including collagen-induced arthritis (CIA) and proteoglycan-induced arthritis (PIA) have been studied on the PD-1 knockout background in order to better uncover the role of this pathway in disease pathogenesis. In PD-1 knockout mice, CIA has been observed to progress more often to severe disease, including increased T-cell proliferation (Raptopoulou et al., 2010; Yang et al., 2016; Zhang et al., 2021b). *Ex vivo* analysis of T-cells isolated from these mice show an abnormal level of antigen-specific T_H_17 cell activation, along with increased secretion of IL-17 (Yang et al., 2016). High levels of IL-17 in the synovium have long been associated with severe RA and are known to be a major driver of disease progression (Chabaud et al., 1999; Lubberts et al., 2004). A look at the underlying mechanism revealed that PD-1 inhibition of PI3K, PKC-θ, and Akt is necessary to limit CIA progression (Yang et al., 2016). Furthermore, treatment of wild-type mice with PD-L1–immunoglobulin to engage PD-1 led to diminished CIA progression, with decreased levels of IL-17 (Wang et al., 2011). This strategy is supported by evidence that PD-L1 knockout mice show more severe progression of PIA (Hamel et al., 2010). By administering soluble PD-1 to mice, which would bind to PD-Ls and block interaction with and engagement of cell-expressed PD-1, CIA progression was accelerated; IL-17 levels were increased, and joint damage was worsened (Zhang et al., 2021b). All together, these data solidify a role for the PD-1 pathway in limiting the progression of murine models of arthritis and suggest that the PD-1 pathway may hold therapeutic promise in the treatment of human RA.

Analysis of PD-1 expression on peripheral T-cells from RA patients demonstrated that PD-1 is expressed at a lower level compared with healthy controls, and furthermore, PD-1 expression inversely correlated with Disease Activity Score 28 (DAS28) (Li et al., 2014). Within synovial samples, PD-1–expressing T-cells and PD-L1–expressing myeloid cells were enriched and correlated with an observed decrease in T-cell proliferation and cytokine production (Raptopoulou et al., 2010; Zhang et al., 2021b). Conversely, another study of T cells isolated from the synovium concluded that while PD-1 expression is high, it is not easily engaged to inhibit (Raptopoulou et al., 2010). It is not surprising then, given these differing findings on PD-1 function in RA that the role of soluble PD-1 (sPD-1) has also yielded inconsistent conclusions. In one study of plasma samples from patients with early and chronic RA, sPD-1 concentrations were found to be increased in both RA patient populations compared with healthy controls, and the concentrations correlated with DAS28 (Greisen et al., 2014). In a separate study of serum samples taken from patients with RA, sPD-1 levels were reported to be lower in those with RA compared with healthy controls (Li et al., 2014). *In vitro*, CD4 T-cells stimulated with sPD-1 secreted TNF-α and IL-6, and the addition of sPD-1 to cocultures of CD4 T-cells and monocytes induced proliferation of both cell types (Bommarito et al., 2017). Additional further work is necessary to establish sPD-1 as a biomarker of RA incidence and severity.

#### Systemic Lupus Erythematosus

SLE is an autoimmune disease driven by loss of self-tolerance leading to activation of autoreactive T-cells and production of pathogenic Abs (Gremese et al., 2020). SLE primarily affects young, female patients. Many organs are involved, including the skin, joints, kidney, heart, and lungs. Both animal and human data support a role for PD-1 in the pathogenesis of this disease (Zamani et al., 2016; Zhong et al., 2021a).

Deletion of the PD-1 gene in C57BL/6 mice led to lupus-like disease onset. During the course of the disease, these mice presented with inflammatory arthritis and glomerulonephritis secondary to infiltration of immune cells and deposition of immune complexes in the end organs (Okazaki and Wang, 2005). NZB/W is a murine model for lupus nephritis, and when these mice were treated with anti–PD-1–depleting Abs, the animals presented with less severe nephritis secondary to a reduction in the number of the CD4 T-cells that expressed PD-1 together with enhancement in the function of CD4 T_REG_ (Wong et al., 2010). Another study showed that the protective effect of CD28 inhibition in this model was associated with upregulation of PD-1 on the surface of T-cells (Laurent et al., 2017). Altogether these models suggest that the PD-1 is needed to prevent lupus disease in the mice and support its role in the pathogenesis of this disease.

**FIGURE 2:**
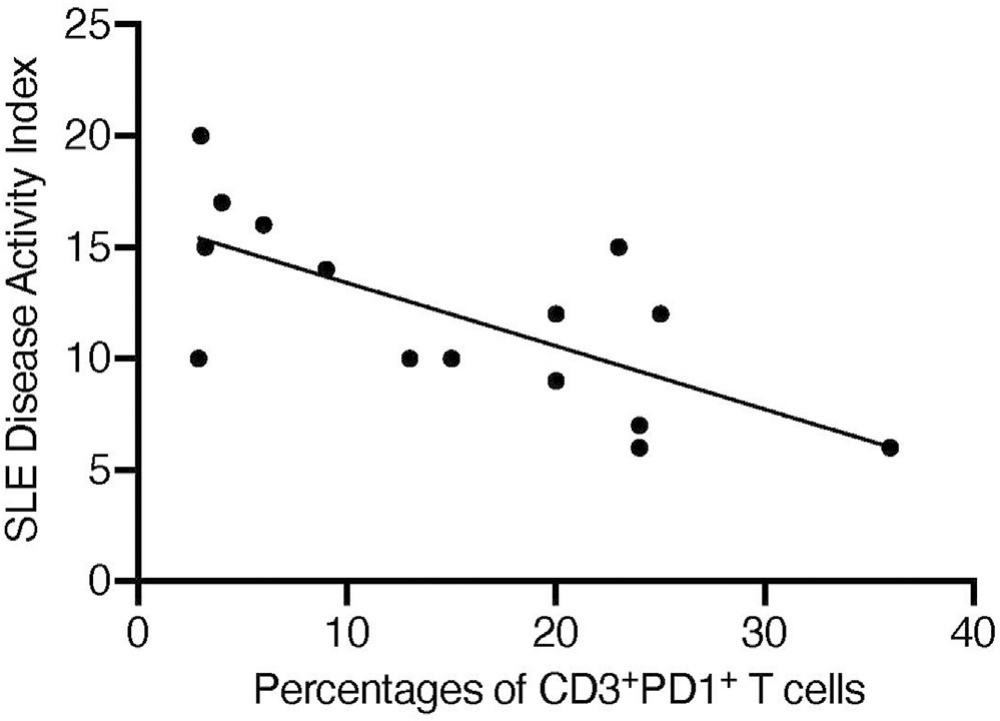
Percentages of CD3^+^PD-1^+^ T-cells inversely correlate with SLE Disease Activity Index. Peripheral T-cells were collected from 15 patients with SLE and the percentages of cells expressing CD3 and PD-1 were calculated by flow cytometry. SLE disease activity was calculated using SLEDAI QxMD and linear regression was analyzed using Prism. *r* ^2^ = 0.487 and *p* = 0.004.

Moving into human data, multiple studies demonstrated that both PD-1 and PD-L1 were expressed at low levels in patients with active SLE and that this was in correlation with disease activity (Dai et al., 2014; Curran et al., 2019). Compared with healthy controls, patients with SLE had less PD-1–expressing CD4 and CD8 T-cells. We analyzed peripheral T-cells isolated from SLE patients and found an inverse correlation between the percentage of CD3 cells expressing PD-1 and disease activity index (Figure 2). Moreover, it was established that when patients with cancer and underlying SLE were treated with anti–PD-1 Abs, many of them experienced a flare of underlying SLE (Zhong et al., 2021a; Tota et al., 2021). In these studies, PD-1 blockade was associated with malar rash, arthritis, serositis, and nephritis (Ramos-Casals et al., 2020; Tota et al., 2021). Mechanistically, it has been suggested that this flare was mediated by the removal of PD-1–expressing T_REG_ cells. Other studies challenged these findings: patients with active childhood-onset SLE exhibited higher frequencies of PD-1–expressing T_REG_, T_EFF_, and naive T-cells under basal conditions than those in the controls and patients with inactive SLE.

Human genetic studies also associated the PD-1 gene, *PDCD1*, with systemic autoimmunity. Linkage analyses between the *PDCD1* and lupus uncovered distinct patterns in different populations (Chua et al., 2015). Specific PD-1 polymorphisms were associated with the development of SLE in an Iranian population but not in a Malaysian population. Other polymorphisms were significantly decreased in patients relative to healthy controls in a Chinese population. For example, the PD1.3 A allele was associated with SLE in Caucasian and Mexican populations, but not in African American and Polish populations. Interestingly, there was no evidence of associations between genetic variants of the PD-1 ligands, PD-L1 or PD-L2, and SLE (Lauwerys and Wakeland, 2005).

To further support the role of PD-1 in systemic autoimmunity, a recent publication by Casanova et al. described a patient with inherited PD-1 deficiency who died of pulmonary autoimmunity (Ogishi et al., 2021). The patient’s T-cells were absent of PD-1, and these cells failed to respond to treatment with anti–PD-1 Abs. In addition to the inability of these cells to secrete IFN-γ, the described patient had an enlarged liver and spleen, along with expansion of the double-negative T-cell population, as seen with other lymphoproliferative autoimmunity. This work emphasized the important role of PD-1 in controlling human self-tolerance and prevention of autoimmunity.

#### Psoriatic Arthritis

A portion of individuals with autoimmune cutaneous psoriasis will develop an accompanying inflammatory arthritis, psoriatic arthritis (PsA) (Liu et al., 2014). By isolating peripheral T-cells from patients with PsA and analyzing PD-1 expression, we found that the percentage of T-cells that expressed PD-1 was higher in PsA patients as compared with healthy controls (Peled et al., 2015). Furthermore, patients with a higher percentage of PD-1–expressing T-cells were found to have a lower DAS28 (Peled et al., 2015). This inverse correlation with the percentage of PD-1–expressing T-cells held true for the incidence of tender and swollen joints, but not for C-reactive protein levels, psoriasis area severity index (Peled et al., 2015). We were then able to challenge T-cells derived from PsA patients with *in vitro* TCR stimulation and PD-1 ligation, finding that PD-1 maintains inhibitory function of IL-2 secretion, Akt phosphorylation, and Rap1 activation (Peled et al., 2015). The conclusions of this study support that high levels of PD-1 expression could be contributing to prevention of inflammatory responses in patients with mild PsA and provide the rationale for engaging the PD-1 pathway in this disease.

#### PD-1 Agonists to Treat Autoimmunity

There is a strong rationale for the development of therapeutic strategies targeting the PD-1 pathway for intervention into autoimmune diseases (Greisen and Deleuran, 2021). One approach to restore peripheral tolerance is to introduce recombinant murine PD-L1 fused with IgG2 Fc (mPD-L1–Fc), and this has been done in the CIA model with resulting decreased T-cell activation and disease severity (Wang et al., 2011). Expression of mPD-L1–Fc in the dextran sodium sulfate–induced colitis model also demonstrated decreased disease severity (Song et al., 2015). Recombinant human PD-L1 fused with IgG4 (hPD-L1–Fc) combined with CD40L blockade in mice delayed the rejection of transplanted islet cells (Gao et al., 2003). These preclinical studies hold exciting promise for the future of autoimmune therapeutics.

### Future Directions

ICIs that target PD-1, its ligands, or CTLA-4 have changed the way cancer is treated worldwide. There are currently 10 anti–PD-1 or anti-PDL1 monoclonal Abs on the market throughout the world, with use in the treatment of 17 different cancers. The future plans for these Abs are to expand combination strategies to target more than one pathway. Over the past few years, the majority of ongoing clinical trials are focused on combinations that include angiogenesis-targeted agents and kinase inhibitors and ICI combinations that overcome resistance.

Strategies to improve efficacy are not the only future goals of the field. As discussed, significant effort must be paid to the separation of efficacy and irAEs. Specifically, by increasing knowledge of predictive factors for the development of irAEs and understanding the underlying mechanisms of inflammation, new combination strategies can be expanded to include therapeutics that address these processes.

Beyond clinical cancer care, as we learn more about the mechanisms underlying irAEs, we can expand the success of PD-1 targeting to include autoimmunity. The same signaling mediators that are responsible for aberrant T-cell activation following PD-1 blockade may contribute to the general pathogenesis of these diseases. The ongoing clinical trials addressing PD-1 stimulation in the treatment of SLE and PsA hold tremendous promise for the field (Grebinoski and Vignali, 2020).
